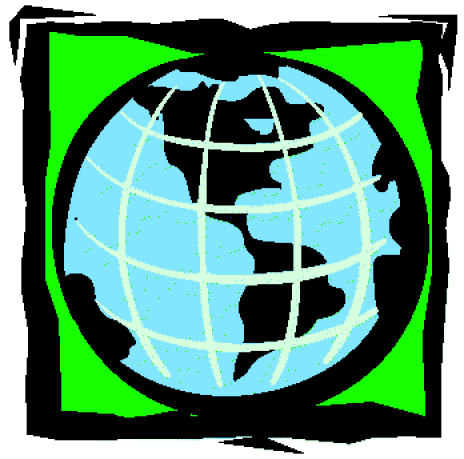# Ethics in Environmental Health

**Published:** 2004-12

**Authors:** Tina Adler

When it comes to the ethics of health research, “there’s been a presumption that ethicists and ethics committees will be in charge and solve ethical problems,” says Ann Cook, director of the National Rural Bioethics Project at the University of Montana in Missoula. More and more, however, environmental health researchers are realizing their need to be directly involved in the ethics questions facing them and their community partners.

Cook describes ethics as “something that everyone in the community has a stake in and needs to know about.” The NIEHS and the National Human Genome Research Institute agree. In 2002 the two institutes launched a grants program called Partnerships to Address Ethical Challenges in Environmental Health, which aims to tackle these issues by promoting community–researcher collaborations.

As part of the larger NIEHS Environmental Justice Program framework, the Partnerships program seeks to remedy the unequal burden borne by socioeconomically disadvantaged persons in terms of residential exposure to greater-than-acceptable levels of environmental pollution, occupational exposure to hazardous substances, and fewer civic benefits such as sewage and water treatment. Chief among ethical concerns for such populations is ensuring that research studies are designed and conducted with the involvement of those being studied rather than regarding them simply as study subjects.

Program grantees, including Cook’s team, receive up to $200,000 annually for five years to investigate environmental ills in a community, survey residents’ attitudes about both local environmental problems and health studies in general, and develop educational campaigns to meet local needs. Grantee teams must include an environmental health scientist, a social scientist or expert on issues such as racial, ethnic, or socioeconomic discrimination, and a representative from a local community organization that works on environmental issues.

A variety of groups, from environmental organizations to schools of public health, receive funding through the Partnerships program. Grantees are now halfway through their projects and ready to discuss some of their findings—and frustrations.

## Defining “Community”

In an effort to develop a series of models for carrying out effective community review of environmental health research, Peggy Shephard, executive director of West Harlem Environmental Action (WE ACT) in New York City, and her colleagues have been listening in on NIH panel discussions between researchers and their community partners. Shephard’s team has also conducted a series of interviews and focus groups with environmental health researchers and their long-term community partners about the workings of such relationships.

One of WE ACT’s preliminary findings is that “we need to stop using the word ‘community,’” says Shephard. The word is repeated so often and in so many contexts that it’s becoming meaningless, she says. In part because “community” has no clear definition among researchers, “we’re coming to the viewpoint that there is never real ‘community consent’ for research,” she says. For example, she asks, is consent achieved when one community group okays a health study, or only when representatives of multiple community groups endorse it? WE ACT is addressing these and other questions—including how to appropriately define “community”—in an upcoming report.

## Ensuring Savvy Study Participants

Researchers at Boston University have rounded up four potentially divergent groups—public health officials, community activists, community residents, and representatives of academe—with the goal of coming to some common understanding of what is involved when scientists embark on a community health study. The team is led by David Ozonoff, an environmental epidemiologist at the Boston University School of Public Health.

What motivated the project, explains project manager and Ozonoff graduate student Madeleine Scammell, is the many calls to university and state health departments across the country from residents concerned about a variety of potential health hazards in their towns. Callers often request a health study, yet when studies are done, communities are often unhappy with the results because of vastly differing expectations about what a health study provides, says Ozonoff. For example, researchers, perhaps preoccupied with the problem of statistical power for small populations, are often stricter than a lay person might expect as to what constitutes positive evidence of an environmental health problem.

At focus groups and during interviews that Ozonoff’s team conducted, residents often reported that it’s tangible evidence of pollution (such as soot on the cars) rather than media coverage that motivates them to take action, says Scammell. Community members are also more concerned than researchers may appreciate about research politics, such as why their town was selected as a study site.

## Teasing Out Interactions

A 200-mile stretch of New York’s Hudson River has achieved the dubious distinction of being one of the country’s largest Superfund sites. Staff at the W. Haywood Burns Environmental Education Center in Albany, where the Hudson and other polluted waterways converge, are investigating what this distinction means for residents of Albany’s poorest neighborhoods.

Led by principal investigator Donna Perry, a registered nurse at the Burns Center, the team and their community partners interviewed residents in 80 primarily African American households to get baseline information on respondents’ health and environment. They found that half of the respondents were smokers. Many had been physically assaulted and reported frequently hearing gunshots near their homes. The smell of gasoline, sewage, and exhaust also was common near their homes. Almost 44% of respondents had breathing problems. Perry and her colleagues are now determining whether the exposure to environmental pollutants in combination with smoking, emotional stress, heredity, lifestyle, and even community zoning decisions may create significant health hazards.

The team is developing health and environmental education materials that are culturally sensitive to the Albany residents they serve. Recommendations for conducting environmental health surveys in urban communities and communities of color are forthcoming, says Perry.

## Countering a Toxic Talisman

Downriver from Albany, Hal Strelnick, a physician at Montefiore Medical Center in the Bronx, leads the South Bronx Environmental Justice Partnership. He and his colleagues are focusing some of their ethics grant dollars on an unusual problem with mercury: members of various religious groups believe that spreading this toxicant around their homes will bring good luck and ward off evil, explains Strelnick.

The ethical challenge of establishing rapport and trust with these groups is complicated; when the New York City Department of Health banned the sale of elemental mercury at the folk pharmacies serving some of these groups, adherents became reluctant to discuss the practice with outsiders. “We wanted to determine if there was a more productive and respectful and ethical way [to educate about mercury],” Strelnick says.

South Bronx residents are not “aware of mercury as an environmental problem unto itself, though they are highly aware of lead, and they understand when you explain that mercury acts like lead in the body,” says Strelnick. The team is partnering with community religious leaders to develop a protocol for educating the public, without panicking them, about the dangers of the ritual use of toxic substances. They are also working on a more general public information campaign on how residents can assess and address community environmental issues.

## Building Trust and Community Capacity for Research

If you ask representatives of community organizations in an area neighboring a prestigious medical school about environmental health and community–researcher relations, you’d better be prepared for a landslide of ideas on how to build effective partnerships. That’s what Mark Farfel, a public health researcher at the Johns Hopkins Bloomberg School of Public Health in Baltimore, Maryland, and colleagues discovered when their Environmental Justice Partnership sought feedback about how to improve the research process.

The partnership uses a participatory model and comprises staff and faculty at the Bloomberg School of Public Health, 11 different East Baltimore organizations, and faculty and students from the Maryland Institute College of Art.

Focus group participants spoke about the poor state of their community’s environment and described negative experiences during research studies, including lack of communication from researchers conducting studies and lack of community involvement. Participants were not entirely negative, however. They agreed that research can be beneficial if the community is involved up front, if the findings are shared with participants and the community at large, and if community–researcher partnerships work to sustain needed programs and policies.

The partnership has followed up by writing grants with board organizations, holding a community fair, and designing educational programs for residents about issues such as lead poisoning. The community board is also working with the Bloomberg School of Public Health to ensure that research in East Baltimore is mutually beneficial.

## Putting Environmental Research on Stage

Communities in North Carolina face environmental contamination from multiple sources, from hog slaughterhouses to wood-laminating industries. Carolyn Crump, a public health scientist at the University of North Carolina at Chapel Hill, and her community colleagues are using theater, along with more traditional educational materials, to open discussion on how health research affects the people who live near pollution hot spots. The team is writing and piloting scripts in the style of Reader’s Theater, in which performers read from a script rather than act out memorized parts. They are also developing facilitator guides that will identify key points for discussion following the performances.

The theater pieces may be performed at community centers, schools, churches, government or other professional offices, conferences, and workshops. The performances are meant to encourage performers and audience members to talk about, among other topics, their understanding of the role of research in identifying environmental hazards, says Crump.

The performances will also document the stories of communities fighting for environmental justice and the experiences of attorneys and researchers who work on environmental health issues. “Cross-disciplinary exchange is one of the main [intended] outcomes of our project,” Crump says.

## Mining the Community Goodwill

Cook’s team in Montana is working with residents in Libby, a mining town in the upper northwest corner of the state. A vermiculite mine that operated in Libby from 1921 to 1990 exposed workers, their families, and the local environment to dangerous levels of toxic amphibole asbestos. “When you are dealing with Superfund kind of issues, communities can get fractured, so we are using information and ethics to bring people together,” says Cook.

Earlier health studies have shown that scientists, health care providers, and Libby residents alike need more information on many issues related to asbestos, including the health risks and health care options. To meet that need, Cook’s team is offering a website (**http://www.umt.edu/Libbyhealth/**) where visitors can read facts that dispel myths about asbestos, download learning activities, and read summaries written in lay language of the legal and scientific issues involved in the Libby case.

The team is also field-testing material designed to help people with asbestos-related disease and other community members understand what a research project is. “In places such as Libby, where there is lots of research going on, you need to clarify what it means to participate in a research project,” says Cook.

To reach out to younger members of the community, the group is developing materials on asbestos and the history of the community for use in local schools. “Schools didn’t discuss [the asbestos problem] with students because it was perceived as a hard topic to talk about,” Cook says.

## Training International Bioethicists

The need for better partnerships between communities and researchers is in no way unique to the United States. The NIEHS also cosponsors, along with several other NIH institutes, projects that address inequities in developing countries.

Developing countries present unique bioethical challenges, says bioethicist Ruth Macklin of the Albert Einstein College of Medicine in the Bronx. For one thing, in countries where many participants are illiterate, written informed consent documents are inappropriate. In addition, she says, the lack of well-trained institutional review boards makes independent ethical review almost impossible. There is also pointed debate about whether foreign investigators need to provide care that is better than or equivalent to what the study participants would normally receive in their country.

To address such issues, Macklin and her colleagues provide seven months of bioethics training every year in Buenos Aires to four Latin American professionals and scholars with experience in studies involving human subjects or research ethics. The training is funded by the John E. Fogarty International Center’s International Bioethics Education and Career Development Awards program, which gives foreign and domestic universities up to $250,000 annually to support international bioethics education for professionals from low- and middle-income countries.

Under the guidance of Macklin and her colleagues, participants take courses in bioethics, attend meetings of different research ethics committees, and prepare a detailed plan for implementing activities in research ethics at their home institutions. Macklin’s recent graduates “are almost without exception engaged in ongoing research or program development in bioethics,” she says.

## A Group Effort

When academic and community groups work together, whether in the United States or abroad, collaborators and participants need to address their long-held assumptions about science, communities, and poverty, ethics grantees say. For example, says Farfel, some African Americans are wary of researchers and their studies in the wake of the infamous Tuskegee Syphilis Study conducted from 1932 to 1972, during which the Public Health Service denied treatment to almost 400 poor African American men who had the disease. Episodes like that lead many would-be study participants to view research as something done *to* them, not *for* them, Farfel says.

Nevertheless, residents are increasingly receptive to this approach of bringing ethics and community participation into all aspects of environmental health research. “Receptive, yes,” says Cook. “And cautious.”

## Figures and Tables

**Figure:**
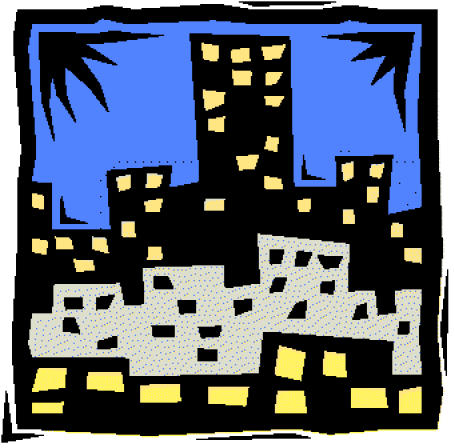


**Figure:**
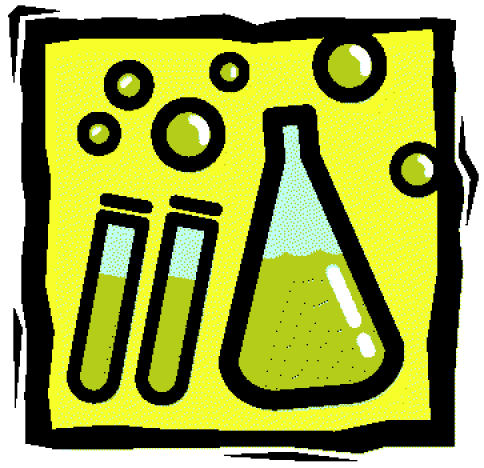


**Figure:**
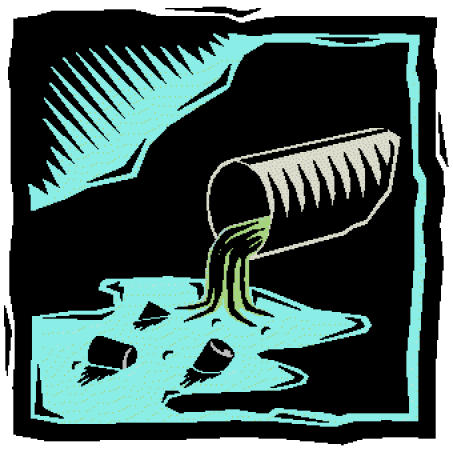


**Figure:**
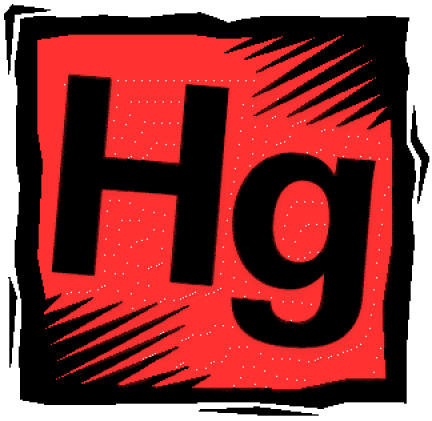


**Figure:**
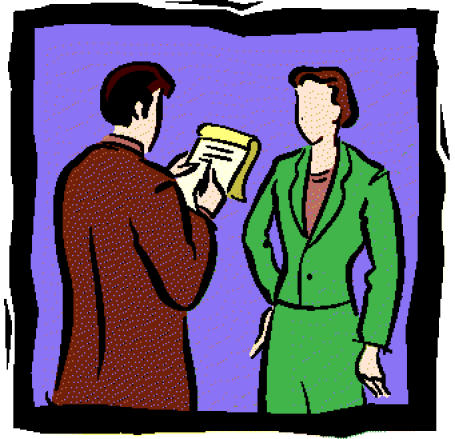


**Figure:**
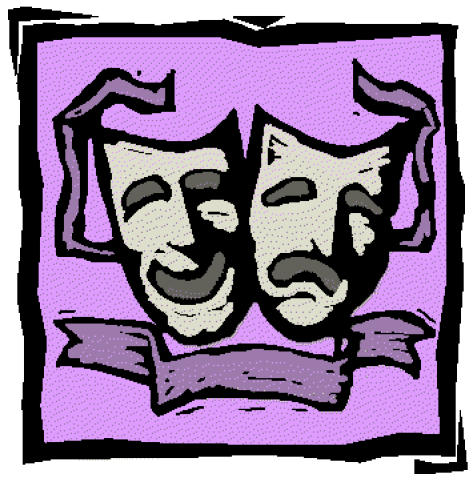


**Figure:**
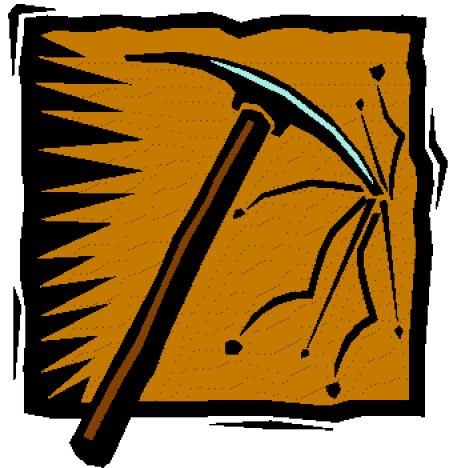


**Figure:**